# Supplementary material for: Inflammation triggers ILC3 patrolling of the intestinal barrier
Source: Nat Immunol. 2022 Aug 23;23(9):1317–23. doi: 10.1038/s41590-022-01284-1 (PMC9477741; doi:10.1038/s41590-022-01284-1)
Supplement: Supplementary file 1 — Supplementary Videos 1–13 [file 41590_2022_1284_MOESM1_ESM.docx]

**Inflammation triggers ILC3 patrolling of the intestinal barrier**

Angélique Jarade, Zacarias Garcia, Solenne Marie, Abdi Demera, Immo Prinz, Philippe Bousso, James P. Di Santo and Nicolas Serafini

**Supplementary Videos**

This file includes: Supplementary video 1 to 13

**SUPPLEMENTARY VIDEOS**

**Supplementary video 1 | Intestinal ILC3s are poorly motile within villi**

Time-lapse video (Z-stack) of intravital imaging of ileum villi in BM chimeric mice. *Actb*^CFP+^ cells, square 1; *Rorc*^GFP+^ ILC3s, square 2 and 3. Scale bar, 50 μm. Z-stack video is representative of 9 different movies obtained in two independent experiments.

**Supplementary video 2 | Intestinal ILC3s are poorly motile and constitutively express *Il22* in ILF**

Time-lapse video (Z-stack) of intravital imaging of ileum isolated lymphoid follicle in BM chimeric mice. Scale bar, 30 μm. Z-stack video is representative of 4 different movies obtained in two independent experiments.

**Supplementary video 3 | Intestinal NKp46^+^ *Il22*^+^ ILC3s are poorly motile within villi**

Time-lapse video (Z-stack) of intravital imaging of ileum villi in *Ncr1*^GFP^*Il22*^TdT^ mice. Nuclei were stained with Hoechst prior to imaging. *Ncr1*^GFP+^*Il22*^TdT+^ ILC3, square 1; *Ncr1*^GFP−^*Il22*^TdT+^ cell, square 2. Scale bar, 50 μm. Z-stack video is representative of 9 different movies obtained in three independent experiments.

**Supplementary video 4 | Intestinal NKp46^+^ *Il22*^+^ ILC3s patrol and monitor the intestine upon acute inflammation**

Time-lapse video (Z-stack) of intravital imaging of ileum villi in *Ncr1*^GFP^ *Il22*^TdT^ mice 5h after flagellin injection. Scale bar, 50 μm. Z-stack video is representative of 13 different movies obtained in three independent experiments.

**Supplementary video 5 | Adaptive immune cells regulate ILC3 patrolling**

Time-lapse video (Z-stack) of intravital imaging of ileum villi in *Rag2^−/−^Rorc*^GFP^*Il22*^TdT^ mice. Nuclei were stained with Hoechst prior to imaging. ILC3s are shown in the green (GFP) channel, *Il22* transcripts are shown in the red (TdT) channel. Scale bar represents 50 μm. Z-stack video is representative of 9 different movies obtained in three independent experiments.

**Supplementary video 6 | T cells suppress ILC3 patrolling**

Two weeks prior intravital imaging of intestine, *Rag2^−/−^Rorc*^GFP^*Il22*^TdT^ mice were adoptively transferred with CFP^+^ T cells. Time-lapse video (Z-stack) of intravital imaging of ileum villi in T-cell reconstituted *Rag2^−/−^Rorc*^GFP^*Il22*^TdT^ mice. ILC3s are shown in the green (GFP) channel, *Il22* transcripts are shown in the red (TdT) channel, T cells are shown in the blue (CFP) channel. Squares number 1 and 2 highlight ILC3s. Scale bar, 50 μm. Z-stack video is representative of 13 different movies obtained in three independent experiments.

**Supplementary video 7 | Acute inflammation overrides T cell control of ILC3 patrolling**

Time-lapse video (Z-stack) of intravital imaging of ileum villi in flagellin-stimulated T-cell reconstituted *Rag2^−/−^Rorc*^GFP^*Il22*^TdT^ mice (5h after injection). Square highlights ILC3. Scale bar, 50 μm. Z-stack video is representative of 10 different movies obtained in three independent experiments.

**Supplementary video 8 | Chemokines are involved in ILC3 patrolling**

Time-lapse video (Z-stack) of intravital imaging of ileum villi in *Rag2^−/−^Rorc*^GFP^*Il22*^TdT^ mice before and after blocking antibodies anti-CXCL12, anti-CXCL16, anti-CCL21, anti-CCL25) injection with Hoechst. Scale bar, 50 μm. Z-stack video is representative of 3 different movies per condition obtained in three independent experiments.

**Supplementary video 9 | Isotypes control have little effect on ILC3 migration**

Time-lapse video (Z-stack) of intravital imaging of ileum villi in *Rag2^−/−^Rorc*^GFP^*Il22*^TdT^ mice before and after isotypes (mouse IgG1 50 µg, rat IgG2a 100 µg, rat IgG2b) injection with Hoechst. Scale bar, 50 μm. Z-stack video is representative of 3 different movies per condition obtained in three independent experiments.

**Supplementary video 10 | The CCL25/CCR9 axis is involved in ILC3 patrolling**

Time-lapse video (Z-stack) of intravital imaging of ileum villi in *Rag2^−/−^Rorc*^GFP^*Il22*^TdT^ mice before and after Hoechst and anti-CCL25 injection. Scale bar, 50 μm. Z-stack video is representative of 3 different movies per condition obtained in three independent experiments.

**Supplementary video 11 | Isotype control has little effect on ILC3 patrolling**

Time-lapse video (Z-stack) of intravital imaging of ileum villi in *Rag2^−/−^Rorc*^GFP^*Il22*^TdT^ mice before and after Hoechst and isotype (rat IgG2b) injection. Scale bar, 50 μm. Z-stack video is representative of 3 different movies per condition obtained in three independent experiments.

**Supplementary video 12 | T/ILC3 competition for CCL25**

Time-lapse video (Z-stack) of intravital imaging of ileum villi in *Ccr9^−/−^* T-cell reconstituted *Rag2^−/−^Rorc*^GFP^*Il22*^TdT^ mice. Scale bar, 50 μm. Z-stack video is representative of 13 different movies obtained in three independent experiments.

**Supplementary video 13 | T/ILC3 competition for CCL25 (anti-CCL25)**

Time-lapse video (Z-stack) of intravital imaging of ileum villi in *Ccr9^−/−^* T-cell reconstituted *Rag2^−/−^Rorc*^GFP^*Il22*^TdT^ mice before and after Hoechst and anti-CCL25 injection. Scale bar, 50 μm . Z-stack video is representative of 4 different movies per condition obtained in three independent experiments.
