# Supplementary material for: Inflammation triggers ILC3 patrolling of the intestinal barrier
Source: Nat Immunol. 2022 Aug 23;23(9):1317–23. doi: 10.1038/s41590-022-01284-1 (PMC9477741; doi:10.1038/s41590-022-01284-1)
Supplement: Supplementary file 2 — Reporting Summary [file 41590_2022_1284_MOESM2_ESM.pdf]

nature portfolio

Corresponding author(s): Nicolas Serafini

Last updated by author(s): 01/07/2022

Reporting Summary

Nature Portfolio wishes to improve the reproducibility of the work that we publish. This form provides structure for consistency and transparency in reporting. For further information on Nature Portfolio policies, see our Editorial Policies and the Editorial Policy Checklist.

Statistics

For all statistical analyses, confirm that the following items are present in the figure legend, table legend, main text, or Methods section.

n/a	Confirmed	
☐	☒	The exact sample size (*n*) for each experimental group/condition, given as a discrete number and unit of measurement
☐	☒	A statement on whether measurements were taken from distinct samples or whether the same sample was measured repeatedly
☐	☒	The statistical test(s) used AND whether they are one- or two-sided<br>*Only common tests should be described solely by name; describe more complex techniques in the Methods section.*
☒	☐	A description of all covariates tested
☐	☒	A description of any assumptions or corrections, such as tests of normality and adjustment for multiple comparisons
☐	☒	A full description of the statistical parameters including central tendency (e.g. means) or other basic estimates (e.g. regression coefficient) AND variation (e.g. standard deviation) or associated estimates of uncertainty (e.g. confidence intervals)
☐	☒	For null hypothesis testing, the test statistic (e.g. *F*, *t*, *r*) with confidence intervals, effect sizes, degrees of freedom and *P* value noted<br>*Give P values as exact values whenever suitable.*
☒	☐	For Bayesian analysis, information on the choice of priors and Markov chain Monte Carlo settings
☒	☐	For hierarchical and complex designs, identification of the appropriate level for tests and full reporting of outcomes
☒	☐	Estimates of effect sizes (e.g. Cohen's *d*, Pearson's *r*), indicating how they were calculated

Our web collection on statistics for biologists contains articles on many of the points above.

Software and code

Policy information about availability of computer code

Data collection	Intravital imaging: Olympus FV31S-SW 2 (Olympus)<br>Flow cytometry: BD FACSDiva 7 and 8 (BD Biosciences)<br>Biomark: Biomark HD Data Collection 4 (Fluidigm)<br>Immunofluorescence: Imager Z.2 (Zeiss)<br>Fluorescence: Fluostar Optima (BMG labtech)
Data analysis	Intravital imaging: Imaris 7.4.2 (Bitplane), Fiji 2 (ImageJ), DiPer (PMID: 25033209)<br>Flow cytometry: FlowJo 10 (TreeStar)<br>Biomark: Fluidigm Real-Time PCR Analysis 4.5.2 (Fluidigm)<br>Fluorescence: BMG labtech software (v2)<br>All: Prism 8 (GraphPad) and Excel 16 (Microsoft)

For manuscripts utilizing custom algorithms or software that are central to the research but not yet described in published literature, software must be made available to editors and reviewers. We strongly encourage code deposition in a community repository (e.g. GitHub). See the Nature Portfolio guidelines for submitting code & software for further information.

March 2021

Data

Policy information about availability of data

All manuscripts must include a data availability statement. This statement should provide the following information, where applicable:

- Accession codes, unique identifiers, or web links for publicly available datasets
- A description of any restrictions on data availability
- For clinical datasets or third party data, please ensure that the statement adheres to our policy

All data sets generated and/or analyzed during the current study are included in this published article. The accompanying source data or supplementary information are available from the corresponding author upon reasonable request. Source data are provided with this paper.

Human research participants

Policy information about studies involving human research participants and Sex and Gender in Research.

Reporting on sex and gender	n/a
Population characteristics	n/a
Recruitment	n/a
Ethics oversight	n/a

Note that full information on the approval of the study protocol must also be provided in the manuscript.

Field-specific reporting

Please select the one below that is the best fit for your research. If you are not sure, read the appropriate sections before making your selection.

☒ Life sciences ☐ Behavioural & social sciences ☐ Ecological, evolutionary & environmental sciences

For a reference copy of the document with all sections, see nature.com/documents/nr-reporting-summary-flat.pdf

Life sciences study design

All studies must disclose on these points even when the disclosure is negative.

Sample size	Sample sizes were derived from initial preliminary experiments and from a principle of repeating all in vivo experiments at least twice to ensure experimental reproductibilty. No statistical methods were used to pre-determine samples sizes. For intravital imaging experiments, external parameters were controlled (temperature, oxygen), experimental bias was limited (no sample preparation besides surgery), multiple XY positions could be simultaneously acquired and the effect size was large – therefore a small sample size (n=3 in general) was powerful enough. For flow cytometry and Biomark experiments that involved sample preparation, sample size was of n=4 minimum (and in general > n=6) and this sample size was powerful enough too.
Data exclusions	No data were excluded from the analyses.
Replication	All the experiments were reliably reproduced as validated by at least two independent experiments.<br>Moreover, two distinct investigators performed the experiments independently. For each technique, two distinct investigators also performed independent data analysis on an identical dataset. All attempts of replication were successful.
Randomization	Experimental groups were not randomized. Mice were grouped according to genotype and all experiments were performed with age- and sex-matched littermates. The sex-specific histocompatibility was observed for adoptive transfer.
Blinding	Investigators were not blinded during the acquisition of the data and analysis because 1) the investigator analyzing data was also collecting it 2) experimental groups could be determined from the data (e.g. images from intravital imaging where adoptive transfer could be deducted from CFP+ T cells that could be visualized on images).

Reporting for specific materials, systems and methods

We require information from authors about some types of materials, experimental systems and methods used in many studies. Here, indicate whether each material, system or method listed is relevant to your study. If you are not sure if a list item applies to your research, read the appropriate section before selecting a response.

Materials & experimental systems

n/a	Involved in the study
☐	☒ Antibodies
☒	☐ Eukaryotic cell lines
☒	☐ Palaeontology and archaeology
☐	☒ Animals and other organisms
☒	☐ Clinical data
☒	☐ Dual use research of concern

Methods

n/a	Involved in the study
☒	☐ ChIP-seq
☐	☒ Flow cytometry
☒	☐ MRI-based neuroimaging

Antibodies

Antibodies used

The following antibodies were used for the study (all commercially available): CCR2 BV421 (SA203G11, Biolegend), CCR6 BV421 (140706, BD Biosciences), CCR7 PE (4B12, eBioscience), CCR9 PE (CW-1.2, eBioscience), CD11b AF700 (M1/70, eBioscience), CD19 PerCP-Cy5.5 (1D3, BD Biosciences), CD127 PE-Cy7 (A7R34, eBioscience), CD3 APC (145-2C11, eBioscience), CD3 eF450 (500A2, eBioscience), CD3 BUV737 (17A2, BD Biosciences), CD44 (IM7, BD Biosciences), CD62L (MEL-14, BD Biosciences), Foxp3 (FJK-16s, eBioscience), Gata3 (TWAJ, eBioscience), active Casp3 (C92-605, BD Biosciences), CD45 BUV395 (30-F11, BD Biosciences), CD45.1 APC-Cy7 (A20, BD Biosciences), CD45.2 APC- eF780 (104, eBioscience), CD45.2 BV711 (104, BD Biosciences), CD45.2 FITC (104, BD Biosciences), CD5 APC-R700 (53-7.3, BD Biosciences), CD5 BV605 (53-7.3, BD Biosciences), CD8α BUV805 (53-6.7, BD Biosciences), CD4 APC (GK1.5, eBioscience), CD90.2 PerCP-eF710 (30-H12, eBioscience), CD90.2 BV605 (53-2.1, Biolegend), CD90.2 FITC (53-2.1, eBioscience), CXCR3 BV421 (CXCR3-173, BD Biosciences), CXCR4 PE (QA16A08, Biolegend), CXCR5 PE (L138D7, Biolegend), CXCR6 PE (SA051D1, Biolegend), KLRG1 BV605 (2F1, BD Biosciences), NK1.1 APC-Cy7 (PK136, Biolegend), NK1.1 BUV395 (PK136, BD Biosciences), NKp46 APC (29A1.4, eBioscience), NKp46 PE (29A1.4, eBioscience), RORgt PE-CF594 (Q31-378, BD Biosciences), TCRβ APC-eF780 (H57-597, eBioscience).
All antibodies were used at 1:200, except for CD90.2 was used at 1:800, and CCR2/CXCR3/4/5/6 were used at 1:100.

Validation

All antibodies are commercially available and are validated on the manufacturer's website. All antibodies were initially tested and titrated before routine use in the lab.

Animals and other research organisms

Policy information about studies involving animals; ARRIVE guidelines recommended for reporting animal research, and Sex and Gender in Research

Laboratory animals

Mice were housed and bred in dedicated in animal facilities of the Institut Pasteur (12h light/dark cycle; 22+/-2°C) . All mice were used on a C57BL/6J background. Eight to fourteen weeks-old male and female animals, except for experiments on BM chimeric mice which nvolved sixteen weeks-old males at the time of analysis.

Wild animals

The study did not involve wild animals.

Reporting on sex

All experiments were performed on eight to fourteen weeks-old male and female animals, except for experiments on BM chimeric mice which involved sixteen weeks-old males at the time of analysis.

Field-collected samples

The study did not involve samples collected from the field.

Ethics oversight

All experiments involving mice were performed according to guidelines issued by the Institut Pasteur Ethics Committee and were approved by the French Ministry of Research (projects dha170001, CETEA 2013-033 and CETEA 17500).

Note that full information on the approval of the study protocol must also be provided in the manuscript.

Flow Cytometry

Plots

Confirm that:

☒ The axis labels state the marker and fluorochrome used (e.g. CD4-FITC).

☒ The axis scales are clearly visible. Include numbers along axes only for bottom left plot of group (a 'group' is an analysis of identical markers).

☒ All plots are contour plots with outliers or pseudocolor plots.

☒ A numerical value for number of cells or percentage (with statistics) is provided.

Methodology

Sample preparation

Small intestine was collected from euthanized mice and placed into cold complete medium: RPMI 1640 GlutaMAX (Gibco) supplemented with 5% fetal calf serum (Eurobio) and 10mM HEPES (Sigma-Aldrich). The mesenteric adipose tissue and Peyer's patches were first pulled out before cutting the small intestine longitudinally and removing feces. Intestinal tissue

was washed in PBS (Gibco) to eliminate mucus, cut into 1-2 cm pieces and intestinal epithelial cells were eliminated by shaking incubation in complete medium containing 5 mM EDTA (Invitrogen) for 20 min at 37°C. Subsequently, the intestinal tissue was minced and incubated twice in a digestion solution containing 0.5 mg Collagenase VII (Sigma-Aldrich) for 15 min at 37°C in a shaking incubator to isolate the lamina propria lymphocytes (LPL). LPL were filtered through a 40-µm cell strainer and kept in complete medium for downstream analysis.

Instrument

All the samples were acquired on a custom-configurated LSR Fortessa (BD Biosciences) or sorted using a custom-configurated FACSAria III (BD Biosciences).

Software

Data were collected with BD FACSDiva 7 and 8 (LSR Fortessa, BD Biosciences) and analyzed with FlowJo10 (TreeStar).

Cell population abundance

The purities of sorted ILC3 were more than 98 %, as determined by flow cytometry analysis on the sample containing purified ILC3.

Gating strategy

For all experiments, FSC-A versus SSC-A gates of the starting cell population were used to identify cells. Next, FSC-H versus FSC-W and SSC-H versus SSC-W gates were used to identify singlets cells. Flexible Viability Dye staining was then used to identify live cells. Positive populations were determined by the specific antibodies staining, which were distinct from negative populations. Specifically, for the analysis of intestinal lymphocytes, we applied the following gating strategies: FSC-A versus SSC-A / FSC-H versus FSC-W / SSC-H versus SSC-W / Live (Flexible Viability Dye−) and then
- CD45.2+ / CD3− CD19− / RorcGFP+ CD127+ for ILC3, CD45.2+ / CD3+ for T cells and CD45.2+ / CD19+ for B cells in BM chimeric mice
- CD45+ / Ncr1GFP versus Il22TdT and notably CD45+ / Ncr1GFP+ Il22TdT+ / CD127+ CD3− / NKp46+ NK1.1−/lo for NKp46+ Il22+ ILC3 in Ncr1GFP Il22TdT mice
- CD45.2+ / RorcGFP+ CD3− / RorcGFP+ CD127+ for ILC3, CD45.2+ / RorcGFP+ CD3+ for TH17/22, CD45.2+ / Il22TdT+ CD3− / RorcGFP+ CD127+ for Il22+ ILC3 in (Rag2−/−) RorcGFP Il22TdT mice
- CD45+ / CD3− CD5− / CD90+CD127+ / NK1.1− for ILC NK1.1−, sub-gated on Ncr1GFP and CCR6 for NKp46+ or CCR6+ ILC3 respectively in Ncr1GFP Il22TdT mice
- CD45.2+ / CD3− CD5− / CD19− / CD90+CD127+ / RORγt+ and sub-gated on NKp46 and CCR6 for NKp46+ or CCR6+ ILC3 respectively, CD45.2+ / CD3+ CD5+ for T cells, and for control populations CD45.2+ / CD3+ NK1.1+ for NKT cells, CD45.2+ / CD19+ for B cells, CD45.2+ / CD3− CD5− / CD19− / CD11c+ for myeloid cells, in C57BL6/J mice (chemokine receptors analysis)
- CD45+ CD11b−/ TCRβ+ CD5+ for TCRβ+ T cells and sub-gated on CD8α and CD4 for CD8α+ and CD4+ T cells respectively
For the sorting of ILC3 subsets, we applied the following gating strategies: FSC-A versus SSC-A / FSC-H versus FSC-W / SSC-H versus SSC-W / Live (Flexible Viability Dye−) / CD45.2+ / CD3− / NK1.1− / KLRG1− / CD90.2+CD127+ and sub-gated on NKp46+ or CCR6+ cells.

☒ Tick this box to confirm that a figure exemplifying the gating strategy is provided in the Supplementary Information.
